# Effect of Thickness and Abutment Type on Masking of Advanced Lithium Disilicate Ceramics

**DOI:** 10.3390/dj14050254

**Published:** 2026-04-27

**Authors:** Vibul Paisankobrit, Boonyanood Boonnamma, Papichaya Intajak, Apirat Ritthiti, Katanyoo Limchaikul, Charnsak Sukajintanakarn, Nuttaphon Kittikundecha

**Affiliations:** Department of Conservative and Prosthodontics, Faculty of Dentistry, Srinakharinwirot University, Bangkok 10110, Thailand; vibul@g.swu.ac.th (V.P.); aoeydentssw@gmail.com (B.B.); papichaya@g.swu.ac.th (P.I.); apiratr@g.swu.ac.th (A.R.); katanyoo@g.swu.ac.th (K.L.); charnsak@g.swu.ac.th (C.S.)

**Keywords:** advanced lithium disilicate, masking ability, implant abutment, titanium anodization

## Abstract

**Objectives**: This study aimed to evaluate the masking ability of different thicknesses of advanced lithium disilicate (ALDS) ceramic used for implant-supported crowns compared to conventional lithium disilicate (LDS) and to assess the influence of their combination with various implant abutment materials. **Methods**: Two types of high-translucency computer-aided design/computer-aided manufacturing (CAD/CAM) glass–ceramics in shade A2 were tested: IPS e.max CAD (LDS) and CEREC Tessera (ALDS). Each material was sectioned into four thicknesses (*n* = 8 per group). Four implant abutments were evaluated: titanium (Ti), yellow-anodized titanium (TiY), pink-anodized titanium (TiP), and white zirconia (Zir). The translucency parameter (TP_00_) and color difference (ΔE_00_) between the glass–ceramic and abutment were calculated using the CIEDE2000 formula. **Results**: Significant differences were observed between 1.0 mm and 2.5 mm thicknesses in all groups except for ALDS on TiY. Both glass–ceramics on TiY and TiP showed lower ΔE00 values than those on Ti, except for 2.0 mm and 2.5 mm ALDS. Additionally, their ΔE00 values were lower than those on Zir. Clinically acceptable ΔE_00_ values occurred for 2.5 mm LDS on TiP, 2.0 mm ALDS on TiY and TiP, and 2.5 mm ALDS on TiY and TiP. ALDS demonstrated lower TP_00_ values than LDS at corresponding thicknesses. **Conclusions**: Greater restoration thickness and titanium anodization improved color masking. Anodized titanium enhanced the glass–ceramic masking ability. ALDS at 2.0–2.5 mm on TiY or TiP and 2.5 mm LDS on TiP achieved clinically acceptable masking, with ALDS showing lower translucency than LDS.

## 1. Introduction

Driven by the trend toward reducing metal usage, dental ceramics have become widely utilized in restorative dentistry [1]. Manufacturers have introduced a broad range of ceramic materials, including glass–ceramics, which consist of a crystallized phase embedded in a glassy matrix. This dual-phase composition enhances both strength and optical properties compared to other ceramic types [2]. The integration of computer-aided design/computer-aided manufacturing (CAD/CAM) technology has further advanced the use of glass–ceramics, allowing clinicians to fabricate restorations for natural teeth and dental implants within a single appointment [3,4]. Despite their excellent optical characteristics that closely resemble natural teeth, the mechanical performance of glass–ceramics remains inferior to that of polycrystalline ceramics [2,5]. Therefore, continuous material innovations have focused on improving both esthetics and strength. In 2021, an advanced lithium disilicate (ALDS) glass–ceramic specifically designed for chairside CAD/CAM systems was introduced under the trade name “CEREC Tessera.” This material is suitable for inlays, onlays, veneers, and crowns for natural teeth and implant-supported restorations. ALDS offers enhanced flexural strength, superior esthetics, and a significantly shorter firing time, requiring only 4 min and 30 s for glazing [6].

Dental implants, recognized for their high success rate and biocompatibility, provide an effective treatment option for replacing missing teeth in both partially and completely edentulous patients [7]. The implant abutment, which connects the implant fixture to the prosthetic restoration, is essential in shaping the transmucosal contour of the final crown [8]. Titanium has long been favored for implant abutments due to its strength, fatigue resistance, biocompatibility, and favorable elastic modulus [9,10]. However, the metallic gray color of titanium can compromise esthetic outcomes, particularly in the anterior region [7,9,10,11]. To improve esthetics, titanium anodization has emerged as a practical surface modification technique that alters surface color by forming an oxide layer through electrochemical reactions [9,10,11,12,13]. The thickness of this oxide layer varies with applied voltage, producing a spectrum of surface colors [11]. This process is safe, cost-effective, and noninvasive. Furthermore, previous studies have reported that yellow- and pink-anodized titanium abutments enhance gingival appearance compared to conventional gray titanium [12,13]. Although anodized titanium mitigates the grayish tone of implant abutments, zirconia has also gained popularity as an alternative material due to its high strength and biocompatibility [14]. Zirconia abutments can improve peri-implant soft tissue esthetics [15] and influence the final color of ceramic restorations [7,10], although their opacity can reduce the natural translucency of restorations.

Given the absence of consensus on the esthetic influence of various implant abutments, particularly in the anterior region, ceramic material selection plays a crucial role in achieving natural-looking outcomes. Previous reports showed that glass–ceramics can effectively mask dark underlying substrates [16,17,18]. ALDS, a novel glass–ceramic, presents as a promising option for esthetic restorations due to its enhanced strength and optical properties. Restorations fabricated from ALDS through CAD/CAM systems are monolithic, eliminating the need for veneering porcelain and reducing the risk of chipping. However, limited data exist regarding the masking performance of ALDS over different implant abutment materials.

This study aimed to compare the masking ability of ALDS with conventional lithium disilicate (LDS) under varying thicknesses and across different implant abutment substrates. Both materials were evaluated in their high-translucency forms, commonly used for anterior esthetic restorations. The null hypotheses were that the glass–ceramic type and restoration thickness would not affect color differences across abutment substrates and that translucency parameters would remain comparable between the evaluated materials.

## 2. Materials and Methods

### 2.1. Specimen Preparation

**Figure 1:**
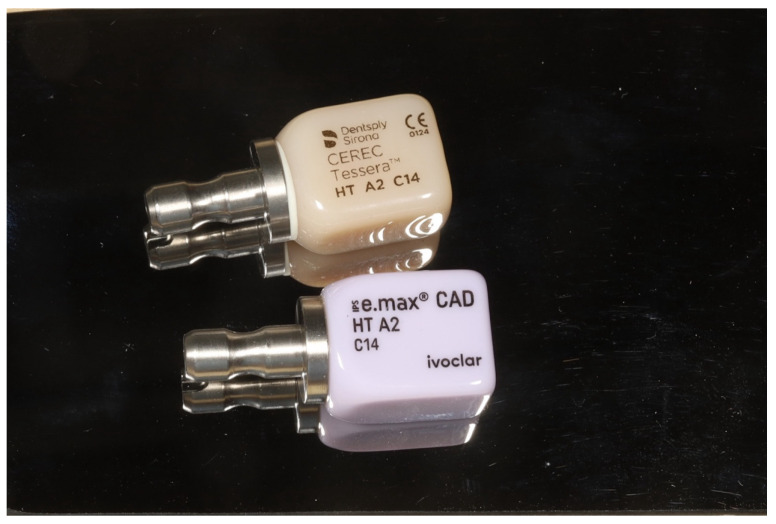
Ceramic tested; advanced lithium disilicate CAD-CAM (computer-aided design/computer-assisted manufacturing) block (top), lithium disilicate CAD-CAM (computer-aided design/computer-assisted manufacturing) block (bottom).

**Figure 2:**
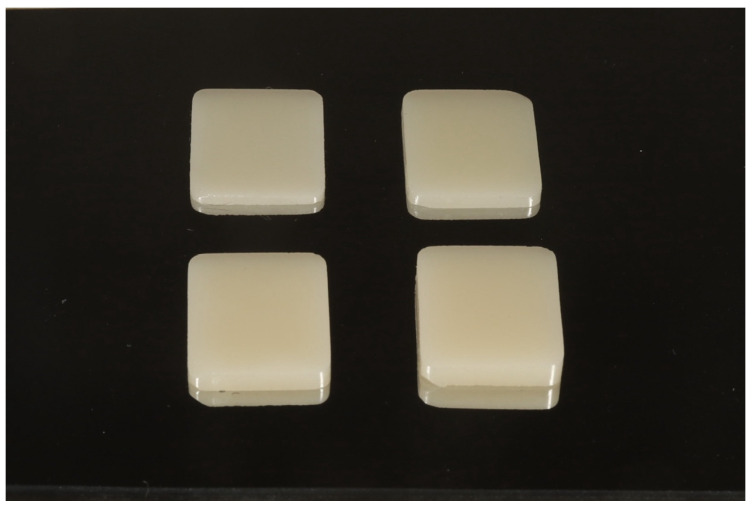
Ceramic specimens in 4 thicknesses.

Two types of square-shaped glass–ceramic specimens (14 × 12 mm^2^) were prepared: LDS (IPS e.max CAD, Ivoclar, Schaan, Liechtenstein) and ALDS (CEREC Tessera, Dentsply Sirona, Hanau-Wolfgang, Germany) (Figure 1). Each material was sectioned from CAD/CAM blocks into four groups (*n* = 8 per group) according to thickness levels of 1.0 mm, 1.5 mm, 2.0 mm, and 2.5 mm (Figure 2). Additionally, specimens with a thickness of 4.0 mm were prepared for the control group, consistent with a previous study [10,19]. Sectioning was performed using a low-speed diamond precision saw (IsoMet 1000 Precision Cutter, Buehler, IL, USA) under continuous water cooling to prevent thermal damage. The specimen surfaces were smoothened using 600-grit abrasive paper on a grinder-polisher under water cooling [5]. Dimensions were verified and standardized using a digital micrometer (Digital Caliper, Mitutoyo, Kawasaki, Japan). The external surfaces were subsequently glazed, followed by firing in a vacuum furnace in accordance with the manufacturer’s instructions [6].

### 2.2. Fabrication of Implant Abutment Substrates

Four types of abutment background substrates were fabricated to simulate implant abutments: titanium (Ti), yellow-anodized titanium (TiY), pink-anodized titanium (TiP), and white zirconia (Zir). The titanium and zirconia substrates were designed as square-shaped blocks (14 × 12 × 2 mm^3^) in digital standard tessellation language format and fabricated in a dental laboratory.

### 2.3. Color Measurement and Spectrophotometric Analysis

Glass–ceramic specimens were cemented onto the abutment backgrounds using a clear-shade try-in paste (NX3 Try-In Gel, Kerr, Brea, CA, USA). A standardized load of 2.5 N was applied for 5 s on each specimen to ensure uniform cementation [20]. The combination of glass–ceramic specimens, try-in paste, and implant abutment backgrounds produced the final color, which was measured inside a dark box. Color measurements were performed using a dental spectrophotometer (Vita Easyshade V; Vita Zahnfabrik, Bad Sackingen, Germany) operating within a spectral range of 400–700 nm. The spectrophotometer tip was positioned in contact with the center of the glazed surface of each specimen. All specimens were measured three times at the same locations, and the spectrophotometer was calibrated before each measurement. A trained researcher conducted all measurements under identical conditions. Color data for the reference specimen (4 mm thick glass–ceramic) and all other specimens were recorded according to the Commission Internationale de l’Éclairage (CIE) parameters L*, a*, and b*. The recorded values were used to calculate the color difference (ΔE_00_) between groups using the CIEDE2000 formula:

$$\Delta E_{00}\;=\;\sqrt{{\left(\left.\frac{\Delta L^{\prime }}{K_{L}S_{L}}\right)\right.}^{2}+{\left(\frac{\Delta C^{\prime }}{K_{C}S_{C}}\right)}^{2}+{\left(\frac{\Delta H^{\prime }}{K_{H}S_{H}}\right)}^{2}+R_{T}\left(\frac{\Delta C^{\prime }}{K_{C}S_{C}}\right){\left(\frac{\Delta H^{\prime }}{K_{H}S_{H}}\right)}^{2}}$$

where ΔL’, ΔC’, and ΔH’ represent differences in lightness, chroma, and hue, respectively. RT is a rotation term accounting for the interaction between chroma and hue differences in the blue region. *S_L_*, *S_C_*, and *S_H_* are weighting functions that adjust the total color difference based on the position of the color pair in the L*, a*, and b* coordinates. The parametric factors *K_L_*, *K_C_*, and *K_H_* serve as correction terms for deviations from reference experimental conditions and were all set to 1 in this study (*K_L_* = *K_C_* = *K_H_* = 1) [7,19]. A ΔE_00_ value of 1.8 was adopted as the clinical acceptability threshold for color difference, as reported by Paravina et al. [21]. The translucency parameter (TP_00_) of the ceramic materials was also evaluated using the same CIEDE2000 formula by calculating color differences between specimens placed over black and white backgrounds [22].

### 2.4. Statistical Analyses

Data were statistically analyzed using IBM SPSS Statistics for Windows, version 25.0 (IBM Corp., Armonk, NY, USA). The Shapiro–Wilk test was applied to assess the normality of the data distribution, and homogeneity of variances was evaluated using Levene’s test. As the ΔE_00_ values demonstrated a nonparametric distribution, the Kruskal–Wallis test (α = 0.05) was used for multiple comparisons involving ceramic material type, ceramic thickness, and abutment material type. Pairwise comparisons within groups were analyzed using the Mann–Whitney U test. For TP_00_ values, which showed a parametric distribution, a one-way analysis of variance (ANOVA) followed by Tukey’s post hoc test (α = 0.05) was applied to compare different glass–ceramic restorative materials at identical thickness levels.

## 3. Results

The mean and standard deviation values of ΔE_00_ are summarized in Table 1. The ΔE_00_ values gradually decreased with increasing specimen thickness across all implant abutment substrates. Clinically acceptable color difference values (ΔE_00_ ≤ 1.8) were observed for the 2.0 mm and 2.5 mm ALDS specimens over TiP and TiY, and for the 2.5 mm LDS specimens over TiP. The Kruskal–Wallis and Mann–Whitney U tests revealed that both glass–ceramic thickness and abutment material type significantly influenced ΔE_00_ values (Table 2 and Table 3). A significant difference (*p* < 0.05) was identified between specimens with 1.0 mm and 2.5 mm thickness for all background types, except for ALDS over TiY. For all glass–ceramic types, ΔE_00_ values over TiY and TiP were significantly lower than those over uncoated titanium, except for ALDS at 2.0 mm and 2.5 mm thicknesses (*p* < 0.05). Additionally, all glass–ceramic types exhibited significantly lower ΔE_00_ values over TiY and TiP compared to zirconia backgrounds (*p* < 0.05).

**Figure 3:**
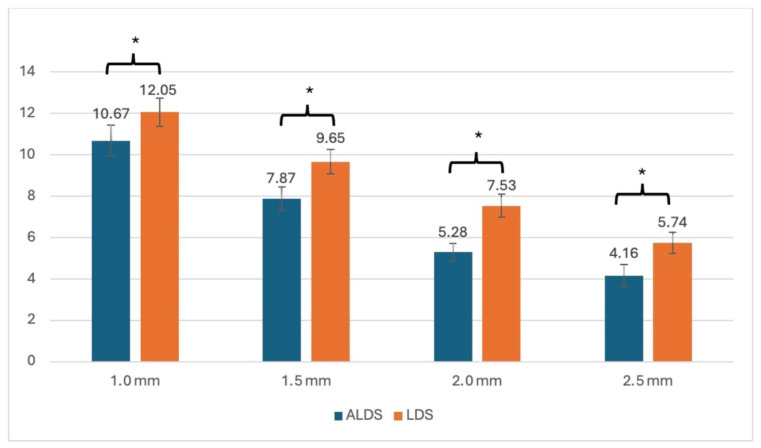
Mean and standard deviation of translucency parameters (TP_00_). Asterisks (*) refer to statistical difference of TP_00_ among each type of ceramic thickness.

Regarding TP_00_, the mean and standard deviation are presented in Table 4. ALDS demonstrated consistently lower TP_00_ values than LDS across all thickness levels. The one-way ANOVA revealed a statistically significant effect of glass–ceramic type (*p* < 0.05). TP_00_ values of ALDS were significantly lower than those of LDS at the same thickness levels (*p* < 0.05) (Table 5 and Table 6, Figure 3).

## 4. Discussion

The findings of this study showed that the type of glass–ceramic restorative material, the thickness of the glass–ceramic, and the type of implant abutment significantly influenced the ΔE_00_ of the specimens. Moreover, the type of glass–ceramic restorative material affected the TP_00_ values. These results led to the rejection of the null hypotheses.

Both ΔE_00_ and TP_00_ values in this study were calculated using the CIEDE2000 formula. Ghinea et al. reported that the CIEDE2000 formula provides greater clinical relevance for evaluating dental ceramics than the CIELab system [23]. Additionally, Gomez-Polo et al. emphasized that CIEDE2000 reflects human visual perception of color differences more accurately than the traditional CIELab (ΔE_ab_) and recommended its use in dental color assessments [24].

Most previous studies on the translucency of dental materials have relied on translucency parameter measurements [2,5,25,26]. The present study also focused on determining TP_00_ values, which quantify translucency differences. A higher TP_00_ value indicates greater translucency, whereas a lower TP_00_ value indicates higher opacity. The translucency parameter serves as an indirect measure of masking ability, as materials with lower translucency can better conceal underlying discolorations [16,22]. The present findings showed that the type of glass–ceramic significantly influenced TP_00_. Gunal and Ulusoy also observed that the translucency parameter varies according to ceramic type, primarily due to differences in material composition. Factors such as crystal structure, chemical composition, crystal content, and particle size determine light transmission and reflection within the material, influencing its translucency–opacity behavior [2]. When comparing material types, ALDS showed significantly lower TP_00_ values than LDS across all thickness levels. LDS contains approximately 70% lithium disilicate crystalline structure [27], whereas CEREC Tessera (ALDS) comprises two crystalline phases, lithium disilicate and lithium aluminum silicate (vigilite), embedded in a zirconia-enriched glassy matrix [6]. The presence of zirconia in ALDS likely contributed to its reduced TP_00_ values, as zirconia contains numerous crystalline particles and a minimal vitreous phase, limiting light transmission and reflection. Consequently, zirconia-rich ceramics exhibit lower translucency. Due to this lower translucency, ALDS demonstrated smaller color difference (ΔE_00_) values than LDS in all tested groups, indicating superior masking performance.

In this study, a statistically significant color difference was observed between the glass–ceramic specimens with thicknesses of 1.0 mm and 2.5 mm. This observation aligns with the findings of Jirajariyavej et al., who reported that increasing the thickness of a ceramic crown reduces the influence of the background shade [19]. However, the present study revealed that the color difference values of ALDS over TiY did not vary significantly across different thicknesses. When comparing the color parameters L*, a*, and b* of the ALDS reference specimen (L* = 72.9, a* = −0.8, b* = 27.8) with those of LDS (L* = 75.3, a* = −1.5, b* = 12.0), the b* value, which represents the blue–yellow color axis, provides additional insight. Positive b* values indicate a more yellow hue, whereas negative values indicate blue. Therefore, the higher b* value of ALDS reflects a more yellowish tone, which enhances its color harmony with TiY across all tested thicknesses.

The color differences between glass–ceramic materials and implant abutment backgrounds across various abutment types and ceramic thicknesses were analyzed in this study using highly translucent ALDS and LDS to minimize the confounding effect of material translucency on color masking. The challenge associated with implant abutment substrates extends beyond the dark appearance of titanium, as the whitish, opaque nature of white zirconia can also influence the final shade of restorations. Despite increased thickness and low TP_00_ values, the findings of this study revealed that certain groups of highly translucent monolithic glass–ceramic fabricated from CAD/CAM blocks for implant-supported crowns may not adequately mask either the grayish appearance of titanium or the whitish tone of zirconia backgrounds. Nevertheless, the present study showed that anodization reduced ΔE_00_ values across specimens. This finding aligns with the results of Farrag et al., who reported that TiY reduced specific color parameter values and overall color differences (ΔE_00_) between LDS and implant abutments [9]. The use of TiY and TiP enhanced esthetic outcomes compared to unanodized titanium and resulted in lower ΔE_00_ values than those observed with zirconia backgrounds. The zirconia specimens used in this study exhibited a white shade with a high degree of opacity, which may have adversely affected color matching between the glass–ceramic restorations and the underlying implant abutments. In contrast, the yellow and pink hues of anodized titanium demonstrated greater similarity to both the glass–ceramic and natural tooth shades, contributing to improved overall color blending between the restoration and the background substrate. In addition to influencing the color difference between the restoration and the underlying abutment, anodization also affects the color relationship between peri-implant soft tissues and the abutment surface. Wang et al. compared color matching between human gingiva and titanium abutments with and without anodization in the anterior region and reported that TiP and TiY exhibited lower color difference values than unanodized titanium [12,13]. Collectively, previous studies and the present findings support the conclusion that the anodization of titanium abutments represents an effective and clinically applicable approach for enhancing esthetic outcomes in the anterior region.

Considering the clinical acceptability thresholds, the ΔE_00_ values of 2.0 mm and 2.5 mm thick ALDS over TiY and TiP fell within the clinically acceptable range (ΔE_00_ ≤ 1.8), whereas the ΔE_00_ values of 2.5 mm thick LDS over TiP also remained within this threshold. Specimens meeting this criterion demonstrated the effective masking ability of glass–ceramic materials against the underlying abutment color. These results indicated that ALDS achieved superior masking of implant abutment color compared to LDS. The results of this study were consistent with those of previous reports suggesting that a ceramic restoration thickness of approximately 2 mm is sufficient to mask the color of underlying backgrounds [17,18]. In addition, ALDS over TiP and TiY, as well as LDS over TiP, exhibit adequate masking capacity at increased thicknesses, particularly in monolithic restorations. These findings are clinically significant for achieving esthetically pleasing outcomes where substrate concealment is essential, such as in the anterior region. Furthermore, while ALDS provides greater opacity, LDS may be more clinically favorable in the anterior region due to its higher translucency, provided adequate thickness is maintained. While patients may prioritize esthetics less in the posterior region, the use of yellow-anodized titanium (TiY) remains advantageous; it contributes positively to the overall shade, offering a beneficial alternative in diverse clinical situations. Based on these principles, the findings of this study provide a valuable guide for clinicians and dental technicians regarding the selection of thickness for highly translucent A2-shade ceramics on various implant substrates, depending on the desired translucency parameter and color masking ability. However, some specimen groups were unable to mask the color of the underlying abutments, likely due to differences between the material’s shade, translucency level and the inherent color of the implant abutment. Both CEREC Tessera and IPS e.max CAD glass–ceramics used in this study are commercially available in various shades and translucency levels, which warrant further investigation to provide clinicians with evidence-based guidance for selecting restorative materials in esthetic regions. Previous research has shown that zirconia is widely used for implant abutments due to its favorable mechanical strength and esthetic shade. Nevertheless, the findings of this study indicated that ΔE00 values of specimens placed over zirconia were higher than those over anodized titanium, and none of the specimen groups effectively masked the zirconia color. The use of white-shade zirconia in this study may have contributed to these results. Studies by Jirajariyavej et al. and Chongkavinit and Anunmana evaluated factors influencing the final color of restorations using both white- and yellow-shade zirconia as abutment backgrounds and compared them with titanium. Their findings revealed that none of the specimen groups successfully masked white-shade zirconia, whereas several groups effectively masked yellow-shade zirconia [10,19]. Consequently, yellow-shade zirconia should be considered in future research as a potential alternative abutment material when titanium is contraindicated.

In addition to the thickness of the glass–ceramic and the type of glass–ceramic or abutment background, several other factors can influence the masking ability of restorations. The try-in paste plays a crucial role in predicting the final color of restorations, as it simulates the optical effect produced by completely polymerized resin luting cement after light activation. A clear-shade try-in paste was selected in the present study to ensure consistent adhesion between the implant abutment and the glass–ceramic interfaces while controlling the optical connection factor. Future investigations assessing the effect of different shades of luting cement on the color outcomes of monolithic glass–ceramic restorations are recommended.

The purpose of this study was to evaluate the translucency parameter and masking ability of a newly developed glass–ceramic material, ALDS, commercially known as CEREC Tessera. The findings demonstrated that ALDS exhibits a more yellowish hue and greater opacity compared to LDS. Additionally, combining ALDS at 2.0 mm and 2.5 mm thicknesses with TiY and TiP resulted in an improved esthetic outcome. Beyond its optical advantages, ALDS possesses favorable mechanical and practical features, including high flexural strength, rapid processing time, and pre-crystallized CAD/CAM block availability, which facilitates simplified shade selection before fabrication. These characteristics highlight ALDS as a promising material for contemporary dental restorations.

This study had certain limitations. Only one shade and translucency level of glass–ceramic materials were evaluated, along with a single shade of try-in paste simulating luting cement. In addition, this study was limited by the fact that it was an in vitro study and the geometry of the natural tooth in the application of the crown on dental implant, luting agent, and aging was not considered, which could bring about a change in the overall masking ability of the all-ceramic restorations. Future research should explore a broader range of ceramic materials with varying translucency levels, multiple shades of luting cement, and other factors which were not involved in this study to better understand their combined effects on the final esthetic outcomes of implant-supported restorations.

## 5. Conclusions

Increasing the restoration thickness over implant abutment substrates and anodizing titanium abutments enhanced the color matching of restorations. TiY and TiP abutments improved the masking ability of the tested glass–ceramic materials. Highly translucent A2-shade ALDS at 2.0 mm and 2.5 mm thicknesses, combined with TiY and TiP abutments, provided clinically acceptable masking of the underlying titanium color, whereas highly translucent A2-shade LDS at 2.5 mm thickness with pink anodizing achieved a similar result. ALDS exhibited lower translucency than LDS and demonstrated superior ability to mask the color of implant abutments.

## Figures and Tables

**Table 1:** Mean and standard deviation of color differences (ΔE_00_).

Type of Glass–Ceramic	Backgrounds	Thickness (mm)			
		1.0	1.5	2.0	2.5
ALDS	Ti	8.24 ± 1.33	3.90 ± 1.81	2.21 ± 0.75	2.38 ± 1.18
	TiY	2.23 ± 1.72	2.06 ± 0.95	1.77 ± 0.80 *	1.74 ± 0.43 *
	TiP	5.27 ± 1.54	2.21 ± 1.80	1.67 ± 1.03 *	1.64 ± 0.39 *
	Zr	8.60 ± 0.65	7.53 ± 0.82	5.27 ± 0.91	3.75 ± 0.89
LDS	Ti	9.98 ± 1.51	6.39 ± 0.74	4.83 ± 0.60	3.93 ± 0.55
	TiY	5.36 ± 0.63	3.53 ± 0.32	2.63 ± 0.72	2.11 ± 0.76
	TiP	7.11 ± 0.75	4.40 ± 0.95	3.41 ± 0.77	1.74 ± 1.06 *
	Zr	11.91 ± 1.02	9.48 ± 1.17	6.80 ± 0.86	5.00 ± 0.42

LDS, lithium disilicate; ALDS, advanced lithium disilicate; Ti, titanium; TiY, yellow-anodized titanium; TiP, pink-anodized titanium; Zir, white zirconia. Asterisks (*) indicate ΔE_00_ values is in clinical acceptable range (ΔE_00_ ≤ 1.8) (*p* < 0.05).

**Table 2:** Median and Interquartile range (IQR) of color difference values in Kruskal–Wallis and Mann–Whitney U tests test for comparison thicknesses for influence of glass–ceramic type and implant abutment substrates.

Glass–Ceramic	Background	Thicknesses (mm)				*p*-Value	Pairwise	
		1.0	1.5	2.0	2.5		Match	*p*-Value
ALDS	Ti	8.42 (2.11)	3.38 (2.50)	1.97 (0.99)	2.1 (2.12)	<0.001	1–1.5	0.001
							1–2.0	<0.001
							1–2.5	<0.001
							1.5–2.0	0.021
							1.5–2.5	0.038
							2–2.5	0.721
	TiY	1.41 (2.20)	1.88 (0.78)	1.86 (0.99)	1.88 (0.85)	0.906	1–1.5	0.645
							1–2.0	1
							1–2.5	0.959
							1.5–2.0	0.645
							1.5–2.5	0.574
							2–2.5	0.645
	TiP	5.38 (1.43)	1.55 (2.53)	1.64 (1.23)	1.58 (0.61)	0.002	1–1.5	0.005
							1–2.0	<0.001
							1–2.5	<0.001
							1.5–2.0	0.645
							1.5–2.5	0.959
							2–2.5	0.959
	Zir	8.76 (1.11)	7.76 (1.64)	5.23 (1.61)	3.66 (0.77)	<0.001	1–1.5	0.028
							1–2.0	<0.001
							1–2.5	<0.001
							1.5–2.0	<0.001
							1.5–2.5	<0.001
							2–2.5	0.005
LDS	Ti	9.97 (2.77)	6.44 (1.43)	5.01 (0.92)	4.09 (1.00)	<0.001	1–1.5	<0.001
							1–2.0	<0.001
							1–2.5	<0.001
							1.5–2.0	<0.001
							1.5–2.5	<0.001
							2–2.5	0.015
	TiY	5.44 (1.00)	3.63 (0.62)	2.65 (1.36)	2.48 (1.36)	<0.001	1–1.5	<0.001
							1–2.0	<0.001
							1–2.5	<0.001
							1.5–2.0	0.01
							1.5–2.5	<0.001
							2–2.5	0.234
	TiP	7.06 (0.85)	4.18 (1.19)	3.46 (1.43)	1.86 (2.07)	<0.001	1–1.5	<0.001
							1–2.0	<0.001
							1–2.5	<0.001
							1.5–2.0	0.05
							1.5–2.5	<0.001
							2–2.5	0.015
	Zir	12.06 (1.68)	9.59 (2.09)	7.03 (1.49)	5.04 (0.63)	<0.001	1–1.5	0.001
							1–2.0	<0.001
							1–2.5	<0.001
							1.5–2.0	0.001
							1.5–2.5	<0.001
							2–2.5	0.001

LDS, lithium disilicate; ALDS, advanced lithium disilicate; Ti, titanium; TiY, yellow-anodized titanium; TiP, pink-anodized titanium; Zir, white zirconia.

**Table 3:** Median and Interquartile range (IQR) of color difference values in Kruskal–Wallis and Mann–Whitney U tests for comparison of implant abutment substrates for influence of glass–ceramic type and thicknesses.

Glass–Ceramic	Thickness (mm)	Backgrounds				*p*-Value	Pairwise	
		Ti	TiY	TiP	Zir		Match	*p*-Value
ALDS	1.0	8.42 (2.11)	1.41 (2.20)	5.38 (1.43)	8.76 (1.11)	<0.001	Ti-TiY	<0.001
							Ti-TiP	0.002
							Ti-Zir	0.721
							TiY-TiP	0.007
							TiY-Zir	<0.001
							TiP-Zir	<0.001
	1.5	3.38 (2.50)	1.88 (0.78)	1.55 (2.53)	7.76 (1.64)	<0.001	Ti-TiY	0.003
							Ti-TiP	0.028
							Ti-Zir	0.001
							TiY-TiP	0.574
							TiY-Zir	<0.001
							TiP-Zir	<0.001
	2.0	1.97 (0.99)	1.86 (0.99)	1.64 (1.23)	5.23 (1.61)	<0.001	Ti-TiY	0.328
							Ti-TiP	0.234
							Ti-Zir	<0.001
							TiY-TiP	0.721
							TiY-Zir	<0.001
							TiP-Zir	<0.001
	2.5	2.1 (2.12)	1.88 (0.83)	1.58 (0.61)	3.66 (0.77)	0.002	Ti-TiY	0.328
							Ti-TiP	0.195
							Ti-Zir	0.065
							TiY-TiP	0.645
							TiY-Zir	<0.001
							TiP-Zir	<0.001
LDS	1	9.97 (2.77)	5.44 (1.00)	7.06 (0.85)	12.06 (1.68)	<0.001	Ti-TiY	<0.001
							Ti-TiP	<0.001
							Ti-Zir	0.028
							TiY-TiP	0.001
							TiY-Zir	<0.001
							TiP-Zir	<0.001
	1.5	6.44 (1.43)	3.63 (0.62)	4.18 (1.19)	9.59 (2.09)	<0.001	Ti-TiY	<0.001
							Ti-TiP	0.002
							Ti-Zir	<0.001
							TiY-TiP	0.015
							TiY-Zir	<0.001
							TiP-Zir	<0.001
	2	5.01 (0.92)	2.65 (1.36)	3.46 (1.43)	7.03 (1.49)	<0.001	Ti-TiY	<0.001
							Ti-TiP	0.003
							Ti-Zir	<0.001
							TiY-TiP	0.105
							TiY-Zir	<0.001
							TiP-Zir	<0.001
	2.5	4.09 (1.00)	2.48 (1.36)	1.86 (2.07)	5.04 (0.63)	<0.001	Ti-TiY	<0.001
							Ti-TiP	<0.001
							Ti-Zir	0.001
							TiY-TiP	0.574
							TiY-Zir	<0.001
							TiP-Zir	<0.001

LDS, lithium disilicate; ALDS, advanced lithium disilicate; Ti, titanium; TiY, yellow-anodized titanium; TiP, pink-anodized titanium; Zir, white zirconia.

**Table 4:** Mean and standard deviation of translucency parameters (TP_00_).

Type of Glass–Ceramic	Thickness (mm)			
	1.0 mm	1.5 mm	2.0 mm	2.5 mm
ALDS	11.45 ± 0.59	8.82 ± 0.94	5.25 ± 0.52	3.17 ± 0.53
LDS	12.05 ± 0.69	9.65 ± 0.59	7.53 ± 0.56	5.74 ± 0.50

LDS, lithium disilicate; ALDS, advanced lithium disilicate.

**Table 5:** One-way ANOVA for translucency parameters (TP_00_) at 1.0 mm and 1.5 mm thicknesses.

	Translucency Parameters (TP_00_) for 1.0 mm Thickness					Translucency Parameters (TP_00_) for 1.5 mm Thickness				
	Sum of Squares	df	Mean Square	F	Sig.	Sum of Squares	df	Mean Square	F	Sig.
Between Groups	7.618	1	7.618	14.736	0.002	12.620	1	12.620	37.286	0.000
Within Groups	7.237	14	0.517			4.739	14	0.338		
Total	14.855	15				17.359	15			

df, degrees of freedom.

**Table 6:** One-way ANOVA for translucency parameters (TP_00_) at 2.0 mm and 2.5 mm thicknesses.

	Translucency Parameters (TP_00_) for 2.0 mm Thickness					Translucency Parameters (TP_00_) for 2.5 mm Thickness				
	Sum of Squares	df	Mean Square	F	Sig.	Sum of Squares	df	Mean Square	F	Sig.
Between Groups	20.183	1	20.183	81.961	0.000	10.081	1	10.081	38.999	0.000
Within Groups	3.447	14	0.246			3.619	14	0.258		
Total	23.630	15				13.699	15			

df, degrees of freedom.

## Data Availability

The original contributions presented in this study are included in the article. Further inquiries can be directed to the corresponding author.
